# Chromatin-Based Transcriptional Reprogramming in Plants under Abiotic Stresses

**DOI:** 10.3390/plants11111449

**Published:** 2022-05-29

**Authors:** Koushik Halder, Abira Chaudhuri, Malik Z. Abdin, Manoj Majee, Asis Datta

**Affiliations:** 1 National Institute of Plant Genome Research, Aruna Asaf Ali Marg, New Delhi 110067, India; khalder@nipgr.ac.in (K.H.); abira5@nipgr.ac.in (A.C.); manojmajee@nipgr.ac.in (M.M.); 2 Centre for Transgenic Plant Development, Department of Biotechnology, School of Chemical and Life Sciences, Jamia Hamdard, New Delhi 110062, India; mzabdin@jamiahamdard.ac.in

**Keywords:** chromatin dynamics, abiotic stress, histone modification, transcriptional reprograming, DNA methylation, epigenetics

## Abstract

Plants’ stress response machinery is characterized by an intricate network of signaling cascades that receive and transmit environmental cues and ultimately trigger transcriptional reprogramming. The family of epigenetic regulators that are the key players in the stress-induced signaling cascade comprise of chromatin remodelers, histone modifiers, DNA modifiers and regulatory non-coding RNAs. Changes in the histone modification and DNA methylation lead to major alterations in the expression level and pattern of stress-responsive genes to adjust with abiotic stress conditions namely heat, cold, drought and salinity. The spotlight of this review falls primarily on the chromatin restructuring under severe abiotic stresses, crosstalk between epigenetic regulators along with a brief discussion on stress priming in plants.

## 1. Introduction

During their entire lifespan, from sprouting to senescence, plants are surrounded by multiple stress factors, both abiotic and biotic, and escape is impossible as they are sessile. So, adaptation to the stress condition for self-protection is the only means of defense in their case. As a response to stress, dynamic and transient alterations have been observed in the complex chromatin network, and consequent changes in transcription are their potential response to stimuli [1,2,3]. Chromatin dynamics or chromatin remodeling fall under the category of epigenetic regulation, and this specific phenomenon imparts considerable flexibility to the plant’s phenotype to adjust to unfavorable environmental conditions [4,5]. Epigenetic regulation of the plant phenotypes belongs to various categories namely histone variants, histone and DNA modifiers, chromatin remodelers and non-coding RNAs with regulatory function. The euchromatin regions responsible for transcription possess trimethylated histone 3 lysine 4 (H3K4me3) and acetylated histone 7 lysine 9/23/29 (H3K9ac/H3K23ac/H3K29ac), but may or may not possess CG/CHG/CHH hypomethylation. The core histones H3 and H4 are acetylated, unravelling the chromatin structure and facilitating transcriptional activation. On the contrary, histone deacetylation returns the open chromatin structure into a closed tangled one, thus hindering transcription [6]. Various environmental stress conditions are the stimuli, and subsequent alterations in histone modification and DNA methylation are the responses leading to the expression of different stress-responsive genes [4,7]. The fine thread joining chromatin dynamics and transcriptional changes during the plant stress response are quite a tangled knot. With further research and accrued evidence, this knot is in the process of untangling with revelations of chromatin modifications back-to-back, finally bringing out the transcriptional stress response [8,9]. Here in this review, we will deal with the wide array of chromatin-based transcriptional reprograming and a glimpse of stress priming in plants under major abiotic stress conditions such as heat, cold, drought and salinity.

## 2. Epigenetic Regulations and Chromatin Modifications in Plants

**Figure 1:**
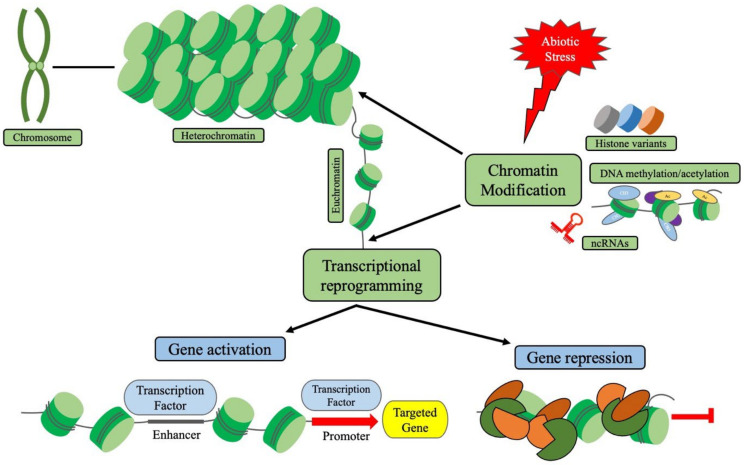
Chromosomes carry all the genetic information required for a plant to survive and the dynamics of chromatin structure (from the highly condensed and repressed heterochromatin state to less condensed and active euchromatin state) regulate the overall gene expression level. Epigenetic regulators such as histone variants, chromatin/histone remodelers, DNA modifiers and non-coding RNAs work in a concerted way to modify the chromatin structure and thereby contribute in regulating gene expressions under abiotic stress conditions in plants.

The eukaryotic chromosome has been classified into two types based on its level of compaction: heterochromatin-the most condensed structure and euchromatin-the less compact form with the ‘beads on a string’ shape. Euchromatin is the accessible form by the transcriptional machinery, and it comprises a central nucleosome unit (two units of histone proteins H2A, H2B, H3, H4) enfolded by approximately 146 bp of linker DNA [10]. These dynamics between active euchromatin and repressed heterochromatin states are modulated by both epigenetic regulators and fundamental processes such as DNA replication, transcription, and repair machinery [11]. Four major epigenetic regulators exist in plants, namely: chromatin remodelers (SWI/SNF, CHD, ISWI and INO80/SWR1) (Table 1), histone modifiers (acetyltransferase, deacetylase, methyltransferase, demethylase, ubiquitylase etc.), DNA modifiers (CHG/CHHCG methyltransferase and demethylase) and regulatory non-coding RNAs (ncRNAs; miRNA, siRNA, lncRNA etc.) (Figure 1) [12]. The majority of genes of histones are intronless genes that reside in the histone clusters in the genome. Histones are synthesized during the S phase of the cell cycle, and their deposition into DNA is facilitated by specific histone chaperon and DNA polymerase [13]. Although there are various histone genes that are present outside of this stipulated cluster in the genome that contain introns, exhibiting different shapes due to alternative splicing phenomena. These forms of histones are called histone variants whose expression and chromatin-deposition are not linked with cell cycles [14]. Some variants have tissue-specific expression, where-as others are expressed uniformly. Some have isoforms named subvariants, some are highly conserved from an evolutionary point-of-view, while others diverge into several lineages [15]. Histone variants play a crucial role in changing the compaction status of chromatin because of their difference in structure and affinity towards DNA. In *Arabidopsis thaliana*, there are four different H2A variants that contribute to the genomic organization. For example, the replacement of H2A variant H2A.X with the H2A.Z can initiate active transcription and can protect further from DNA hypermethylation [16]. The other variant H2A.W works alongside the H3K9me2 and DNA methylation and is required for heterochromatin condensation [17]. The only structural difference between *Arabidopsis thaliana* H3 variants H3.1 and H3.3 are the presence of four extra amino acids [18], but it differs greatly in terms of their mode of actions. As opposed to H3.3, H3.1 is associated with the non-transcribing regions of the genome, replacing it with H3.3 triggers developmental reprogramming [19,20]. *Arabidopsis thaliana* linker histone (H1) stable variants H1.1, H1.2 play critical role in chromatin compaction alongside the most divergent and dynamic variant H1.3. It has the same binding affinity towards the heterochromatin regions as H1.1, H1.2 and is directly required in abiotic stress induced DNA methylation process [21,22]. Recruitments of histone variants in accordance with various abiotic stresses stipulate the direct link between stress signals with the chromatin reassembly and further transcriptional reprogramming, which will be discussed in depth below.

**Table 1:** List of four major chromatin remodeller family and their structural details.

Chromatin Remodelers (Family)	Domains	Subunits	Reference
Switching defective/Sucrose nonfermenting (SWI/SNF)	HELICc, DExx HSA, Bromo	BAF, PBAF	[23]
Chromodomain, Helicase, DNA binding (CHD)	HELICc, DExx, Chromo	CHD1, CHD2, CHD3, CHD4, CHD9, NuRD subunits	[24]
Imitation switch (ISWI)	HELICc, DExx, SANT, HAND, SLIDE	CERF, RSF, ACF, NURF, CHRAC, NoRC, WICH, b-WICH	[24]
Inositol requiring 80 (INO80/SWR1)	HELICc, DExx, HSA	Tip60/p400, INO80, SRCAP	[25]

## 3. Chromatin-Based Transcriptional Reprogramming

### 3.1. Under Heat Stress

**Figure 2:**
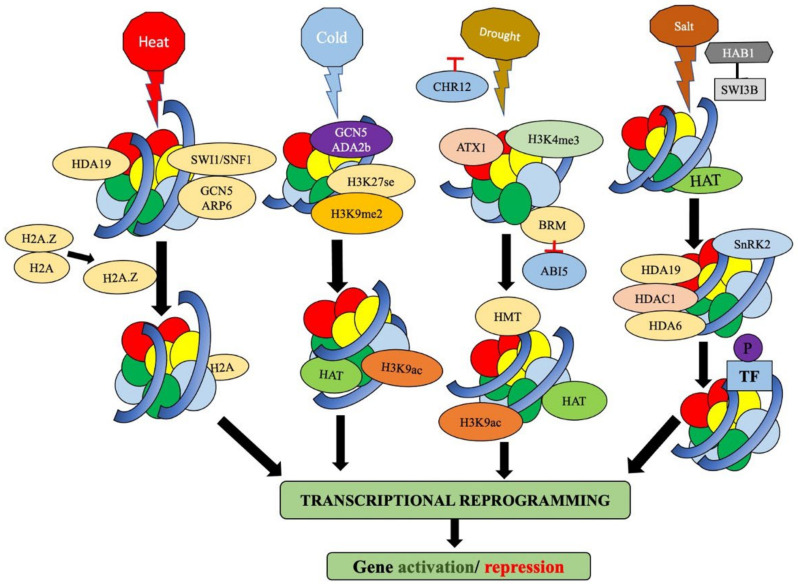
Chromatin dynamics under abiotic stresses such as heat, cold, drought and salinity in plants. During heat stress switching defective/sucrose nonfermenting (SWI1/SNF1) interacts with GCN5, ARP6 resulting in detachment of H2A.Z, facilitating downstream transcriptional reprograming. Transcriptional activator ADA2b, under cold stress, interacts with Arabidopsis GCN5 to boost up the HAT activity, which further results in transcriptional activation. During drought stress, the receptors first inactivate the Chromatin remodeling 12 (CHR12) and BRM. Inhibition in BRM activity further inhibits ABI5, which triggers ABA biosynthesis. Under excess saline conditions HAB1 and SWI3B cannot interact and bind with each other, leading to the activation of SNF1-related kinase (SnRK2) and subsequent phosphorylation of transcription factors leading to gene expression.

Epigenetic regulations including chromatin dynamics associated with abiotic stress conditions are employed by plants to adapt to its sorrounding environment (Table 2; Figure 2). The main pathway of epigenetic regulation to combat stresses such as heat involves DNA methylation, ATP dependent chromatin remodeling, histone modifications, long non-coding RNAs etc.[26,27,28,29]. All the above epigenetic modifications fine-tune the gene expression of heat-responsive genes to handle stress deftly [27]. In *Arabidopsis thaliana* the heat-responsive genes *HSFA3* and *UVH6* show transcriptional activation, which is facilitated and mediated by histone acetyltransferase GCN5 and this happens through the acetylation of H3K9 and H3K14, located in the promoter [30]. *Arabidopsis thaliana* have been potentially used as a model plant for studying epigenetic regulation of heat-responsive genes. There the Anti-Silencing Function1, a well-conserved histone chaperone (ASF1) is quite active in response to heat stress. The homologous genes (*AtASF1A*, *AtASF1B*) play a major part in the activation of gene transcription. The mutant line, *Atasf1ab*, produced in *Arabidopsis thaliana* displayed impaired gene function of many genes, namely, Heat Shock Protein (HSP) genes such as *Hsp17.6B-C1*, *Hsp 17.6A*, *Hsa32*, *Hsp70* and *Hsp 101* along with the HEAT SHOCK FACTOR (HSF) gene *HsfA2*, rather than *HsfB1* is severely paralyzed in the *Atasf1ab* mutant in respect to the wild type variety. The major finding of this experiment was that AtASF1A/B proteins are placed on the chromatin and are highly enriched which is a part of the process of nucleosome deletion and at the same time RNA polymerase II accretion in the promoter regions and coding sites of *HsfA2* and *Hsa32* with *HsfB1* being left aside [31]. An interaction between HD2C deacetylase and BRM possessing SWI/SNF chromatin remodeling complex (CRC), via the SWI orthologue SWI3B, has been experimentally established in the universal model plant *Arabidopsis thaliana*, where HD2C also has a role as a regulator in the plant’s response to heat stress. In this whole process, the heat-activated genes, namely, *HSFA3* and *HSP101* significantly lowered the H4k16ac levels in the coding sites [32]. In a totally opposite phenomenon, it has been noticed that *HSFA2*, the heat-induced transcription factor, turns on the H3K27me3 demethylase RELATIVE OF EARLY FLOWERING6 (REF6) directly, which in turn turns off *HSFA2*, that is genetically transmitted through progeny as a thermomemory [33]. Once the plants become used to heat stress, they prepare themselves for recurring heat stress events in their lifespan. There are heat stress memory-related genes *APX2* and *HSP18.2*. The transcription factor HSFA2 links up directly with the promoter of the above two memory genes, which leads to a sustained collection of H3K4me2/3 at the gene loci of the two memory genes and remains as a potential transcription memory during concurrent phases of heat stress [34,35]. Responding to the stimuli of drastically altering ambient temperature, *Arabidopsis thaliana* behaves in a typical way, while carrying out pivotal processes such as alternative splicing and flowering. H3K36 trimethylation mediated by histone methyltransferase SET DOMAIN GROUP8 (SDG8) and SDG6 is a major activity in response to heat [36]. *Arabidopsis thaliana* behaves strangely during vegetative growth. Its imprinted *SDC* gene reveals a strange but significant role in the recovery process after exposure to heat stress. This gene is inactivated by DNA methylation and contrarily is activated by heat stress, hence proving the theory [37]. On the other hand, ISWI genera of chromatin remodelers (CHR11/17) and SWI/SNF (BRM) come into action and take part in nucleosome remodeling, where the *Arabidopsis thaliana* FORGETTER1 (FGT1) links up with the nearest promoter of *HSA32* and *HSP18.2/22.0* genes and establishes sustainable induction of the above genes post-heat stress acclimatization [38].

**Table 2:** Chromatin dynamics associated with abiotic stress conditions such as heat, cold, drought and salinity in plants.

Species	Stress	Chromatin Modifications	Genes Involved	Reference
*Arabidopsis thaliana*	Heat	H3K4me2/3	*APX2* and *HSP18.2*	[34,35]
*Arabidopsis thaliana*	Heat	H3K36me3	Alternative splicing related genes	[36]
*Arabidopsis thaliana*	Heat	H3K9/14ac	*HSFA3*, *UVH6*	[30]
*Arabidopsis thaliana*	Heat	H3K56ac	*HSFA2*, *HSP32*	[31]
*Arabidopsis thaliana*	Heat	H3K16ac	*HSFA3*, *HSP101*	[32]
*Arabidopsis thaliana*	Heat	H3K27me3	*HSFA2*	[33]
*Arabidopsis thaliana*	Heat	Chromatin remodeling	*HSA32*, *HSP18.2/22.0*	[38]
*Arabidopsis thaliana*	Heat	5-mC in promoter	*At3g50770*	[39]
*Arabidopsis thaliana*	Cold	Chromatin remodeling	Stimuli-responsive genes	[40]
*Oryza sativa*	Cold	H3K9/14/27ac	*OsDREB1b*	[41]
*Arabidopsis thaliana*	Cold	H3K9/14ac	*RD29A*, *COR15A/47/78*	[30]
*Musa acuminata*	Cold	H3/H4ac	*MaFADs*	[42]
*Solanum tuberosum*	Cold	H3K4/27me3	Cold-responsive genes	[43]
*Brassica rapa*	Cold	5-mC in promoter	*BramMDH1*, *BraKAT2*, *BraSHM4*, *Bra4CL2*	[44]
*Oryza sativa*	Cold	5-mC in promoter	*OsOST1* (*Os03g0610900*)	[45]
*Arabidopsis thaliana*	Cold	5-mC in promoter	*DREB1A*	[46]
*Arabidopsis thaliana*	Cold	H3K4me3	*WRKY70*	[47]
*Arabidopsis thaliana*	Drought	H3K9ac, H3K4me3	*RD29a*, *AtGOLS2 RD20*, *ProDH*	[48]
*Populus trichocarpa*	Drought	H3K9ac	*PtrNAC006*, *PtrNAC007*, *PtrNAC120*	[49]
*Arabidopsis thaliana*	Drought	H3K4me3	*OST1*, *ABF3*, *ATHB7*, *ERD1*	[50]
*Arabidopsis thaliana*	Drought	H3K4me3	*LTP3*, *LTP4*, *HIPP2.2*	[51]
*Arabidopsis thaliana*	Drought	H3K27ac	*AtAREB1*	[52]
*Arabidopsis thaliana*	Drought	H3/H4ac	*ROP6/10/11*	[53]
*Hordeum vulgare*	Drought	H3K4me3, H3K9me2	*HSP17*	[54]
*Arabidopsis thaliana*	Drought	H3K9ac	Dehydration-related genes	[55]
*Zea mays*	Salinity	H3K9ac	*ZmEXPB2*, *ZmXET1*	[56]
*Arabidopsis thaliana*	Salinity	H4ac, H3K27/36/56ac, H3K9me2	*KIN2*, *ERF4/5/6/11*, *STZ*	[57]
*Oryza sativa*	Salinity	H3ac	*LEA1*, *SOS1*	[58]
*Arabidopsis thaliana*	Salinity	H3ac	*NCED4*, *ABI5*, *NAC016/019*, *GA20 × 7*, *LEA4_2*, *P5CS1*	[59]
*Arabidopsis thaliana*	Salinity	5-mC, H3K9me2, H3K9ac	*ROS1*, *APUM3*, *UVH2/5/8*, *MSH6*, *DRB2*, *MOS6*	[60]
*Glycine max*	Salinity	H3K4me3, 5-mC, H3K9ac	*Glyma20g30840*, *Glyma11g02400*, *Glyma08g41450*	[61]
*Arabidopsis thaliana*	Salinity	H3K4me3	*P5CS1*	[62]
*Ricinus communis*	Salinity	H3K4/27me3	*RSM1*	[63]
*Arabidopsis thaliana*	Salinity	H2Bub	*IBR5*, *MKP1*, *PTP1*, *PHS1*, *DsPTP1*	[64]

During the RNAi-mediated gene silencing process, siRNAs, typically 20–30 nucleotides long, induce epigenetic modifications such as DNA cytocine/histone methylations in plants, fungi and metazoans [65]. These epigenetically active siRNAs directly dependent on the RNAi machineries such as Dicer (DCL), which converts long double-stranded RNAs into siRNAs, and Argonaute (AGO) proteins: the slicer component [66] of the RNA-induced silencing complex (RISC), which is also involved in RNA-guided chromatin modification. The primary epigenetic pathway in plants is RNA-directed DNA methylation (RdDM), which was first detected in RNA virus infected plants [67,68]. It is prevalent in angiosperms and is distinct from other siRNA-mediated epigenome modification, as it requires special transcriptional enzymes RNA Polymerase IV and V [69]. In the nucleus, transcripts from DNA Polymerase IV are first incorporated into long dsRNAs, which are then processed into siRNAs by DCL3 and exported to the cytoplasm. There, it becomes loaded onto AGO4 and imported back to the nucleus, where it drives the targeting of nascent scaffold transcript from DNA polymerase V. This targeting allows de novo methylation of cytosine by DNA methyltransferase rendering transcriptional silencing of the genomic loci (transposons and repetitive DNA) transcribed by DNA polymerase V [70,71]. In *Arabidopsis thaliana*, the *At1g34220*, *At1g29475*, and *At1g07590* genes and auxin-responsive genes are transcriptionally reprogrammed by RdDM factor NRPD2, which is considered as the second-biggest subunit of PolIV and PolV [28]. The main participants which have tight control of the RdDM pathway of *Arabidopsis thaliana* under heat stress are the biggest subunits of PolIV (*NRPD1*) and PolV (*NRPE1*) genes, are observed to be upregulated while combating heat stress and simultaneously the expression of the *At3g50770*, the heat-induced gene, shows subdued promoter methylation [39]. Heat-induced DMRs in bok choy are generally found in the vicinity of the transcription start and end regions of the gene-related zones and betray the phenomenon of position-dependent transcriptional silencing [72].

### 3.2. Under Cold Stress

Plants are generally subjected to two types of cold stresses namely chilling and freezing. Epigenetic regulation comes into action via histone modifications and DNA methylations in plants during cold acclimatization and vernalization [73]. Two types of mechanisms are followed for cold tolerance in rice and *Arabidopsis thaliana*. In rice chromatin remodeling via histone H3 acetylation is the main event during cold stress response, as this is of primary necessity to activate cold-inducible genes in the rice genome such as *OsDREB1b* [41], while in *Arabidopsis thaliana*, Trichostatin A, a histone deacetylase inhibitor and 5-Aza-2′-Deoxycytidine, a DNA methylation inhibitor modify and change the expression of all the genes induced by chill or freeze strengthening its stress tolerance capacity [74]. The expression of *COR* genes in *Arabidopsis thaliana* is negatively regulated by the POWERDRESS (PWR)-HOS15-HOS2C complex via repressive chromatin structure and histone deacetylation [75,76]. On the other hand, the *COR* genes (*RD29A*, *COR15A/47/78*) are activated due to the degradation of the histone deacetylase HD2C by the PWR-HOS15 complex during cold stress conditions through H3 acetylation and the non-restrictive chromatin structure. It has been observed that there is an elevated level of transcription of ω-3 fatty acid desaturase genes (*ω-3MaFADs*); in cold-treated banana fruits, which corresponds to elevated levels of H3 and H4 acetylation inside promoters of *ω-3MaFADs*. Another interesting fact is that the transcription of *ω-3MaFADs* is negatively regulated by the transcription factor MaMYB4 with the assistance of MaHDA2, a histone deacetylase [42]. In cold-stored potatoes, which is the main requirement in wholesale and retail trade, the transcription of the cold-induced active genes is of primary necessity. Here, H3K4me3 and H3K27me3, the bivalent histone modifications, facilitate the smooth accessibility of the chromatin network and the essential regulatory proteins necessary for the transcription of the active genes of such type of stored potato [43]. It has been reported that in *Arabidopsis thaliana*, a SUMO E3 ligase is encoded by Arabidopsis SAP and MIZ1 domain-containing ligase1 (SIZ1) and handles various types of stresses. This SIZ1 is a zinc finger motif (C_4_HC_3_), also known as the plant homeodomain finger, or PHD finger. This finger recognized trimethylated histone (H3K4me3). PHD and ATX interact among themselves and mediate histone methylation, negatively regulating the function of ATX. It was also observed that *WRKY70* was up-regulated in cold stress, and simultaneously, H3K4me3 accumulation took place in significant amounts in WRKY70 promoter [47]. In cold-acclimated bok choy, there lies a classical example of promoter demethylation, where the genes *Bram-MDH1*, *BraKAT2*, *BraSHM4*, *BraSHM* and *Bra4CL2* are differentially methylated [44]. Similarly in rice, the gene that participates to combat cold (*OsOST1*, *Os03g0610900*) via the ICE-CBF-COR route, also demonstrates elevated gene expression, which is linked once again with promoter demethylation [45]. A drastically opposite phenomenon was noticed in *Arabidopsis thaliana* ice1-1 mutant. Here, the *DREB1A* gene, whose expression is generally induced by cold is repressed by the phenomenon of hypermethylation, generally transgene-induced located in the DREB1A promoter [46]. Gene transcription is boosted in *Arabidopsis thaliana* by the ARGONAUTE1 (AGO1) as it interacts with the cold stress-responsive genes via small RNAs and other chromatin remodeling complexes such as SWI/SNF [40].

### 3.3. Under Drought Stress

Drought stress tolerance and recovery by plants involve significant and dynamic chromatin alterations which control transcription regulation in turn [77,78,79]. Maize plants are highly adept at modulating their behavior accordingly to adapt, recover and eventually survive drought stress. In many stress-responsive genes such as *ZEP1*, *NCED6*, members of WRKY, NAC and AP2/EREBP transcription factor families, the difference in the transcript levels continue to remain even after the recovery from stress has been completed. Many genes (*MADS4* and *MADS15*) which do not directly take part in stress response were also identified in maize. They recognized the signals and stored them in memory for a much later response [79]. Similar to maize, even in *Arabidopsis thaliana*, chromatin dynamics happen through the eukaryotic marks H3K9ac and H3K4me3 [8,80]. When the *Arabidopsis thaliana* plant is subjected to water deficit, the corresponding response-related genes are either upregulated or downregulated, and significantly altered levels of H3K4me3 play the major role, while H3K4me1 and H3K4me2 play a minor role [50,81,82]. In plants, some genes are called memory genes, and the dehydration-related memory genes of *Arabidopsis thaliana* are *LTP3*, *LTP4* and *HIPP2.2*. These genes are highly activated during concurrent phases of drought, and the elevated levels of H3K427me3 and PolII in the above genes are also associated when the plant recovers from the initial stress phase [51]. Various drought- inducible genes such as *RD20*, *RD29a*, and *AtGOLS2* and recovery-inducible genes such as *ProDH* are activated or repressed via chromatin dynamics through these eukaryotic marks [48]. The activation of certain drought-tolerant genes with revised status of epigenetic modifications is the key to drought tolerance in plants, and this activation is catalyzed by specific transcription factors, but their molecular mechanism is still an enigma. In *Populus trichocarpa*, the enrichment of acetylated lysine residue 9 of histone H3 (H3K9ac) was thoroughly studied along with its linkup with the transcriptomes. It was very clear that the promoter based abscisic acid-responsive element (ABRE) motifs of genes (*PtrNAC006*, *PtrNAC007*, *PtrNAC120*) that perceive and respond to drought stress, not only activate these above genes but are also responsible for H3K9ac and PolII amplification with the assistance of histone acetyltransferase unit ADA26-GCN5 [49]. In the era of genetic engineering and genome editing, there is immense potential for editing these drought-tolerant genes for enhanced activity. The drought tolerance capacity of *Arabidopsis thaliana* can be improved by CRISPR activation (CRISPRa) system. Here, the CRISPR/dCas9-histone acetyl-transferase1 (HAT1) complex targets AREB1, and its expression is upregulated via amplification of H3K27ac at the promoter site [52]. ABA signaling plays a key role in drought tolerance in plants, and in *Arabidopsis thaliana*, it is noticed that the transcription factor MYB96 and histone deacetylase HDA15 act hand-in-hand to suppress the *RHO GTPASE OF PLANTS* (*ROP*) group gene expression (*ROP6/10/11*) by lowering the acetylation of H3 and H4 at the promoter sites [53]. A dip in the H3K9 acetylation in the promoter regions of drought and salinity-responsive genes in *Arabidopsis thaliana* takes place, and thus the histone deacetylase HDA9 enzyme makes the plant susceptible to both the above abiotic stress types [55]. HDA9 also directly communicates with the ABA INSENSITIVE (ABI4) transcription factor during drought to down-regulate the gene expression of *CYP707s* via histone deacetylation [83,84]. In barley plants subjected to drought stress, alterations in the coding regions of the gene *HSP17* take place by the increase and decrease in H3K4me3 and H3K4me2 modifications respectively for the response activity [54].

In different rice genotypes, drought stress response takes place via alterations in DNA methylation throughout the whole genome, which is also linked up with differential transcription and these changes are genotype, development and tissue-specific [85,86,87]. In plants such as *Gossypium hirsutum* (cotton), *Eucalyptus globulus*, *Citrus sinensis* and rice the altered levels of DNA-methylation are brought back to almost normal level at the time of recovery. These types of DNA-methylation are also noticed in numerous phytohormone-linked genes which come into action during the water deficit response [86,88,89,90]. This phenomenon (genome-wide DNA methylation) is an intense response on the part of *Arabidopsis thaliana* to water deficit and the significant alteration takes place at different loci inside the promoters of the corresponding stress response genes [91]. Still, a lot of experimentations are in progress in different labs to establish a scientific link between site-specific DNA methylation and the corresponding transcriptional alterations of the drought-responsive genes. Studies have revealed that dehydration-linked epialleles in the DNA methylome are extremely minimal, and a conclusion cannot be drawn regarding the gene expression under dehydration stress acting across multiple generations [92].

### 3.4. Under Salinity Stress

Similar to the responses of plants to the above-discussed stress conditions, salinity stress too induces common responses such as histone modification and alterations in DNA methylation. These responses to the stress stimulus bring around a noticeable change in the chromatin organization and dynamics which eases the locus-specific gene expression in plants [78]. Studies conducted for a few filial generations of saline-stressed *Arabidopsis thaliana* revealed that the expression of genes such as *SUVH2/5/8*, *ROS1*, *MSH6*, *APUM3*, *MOS6* and *DRB2* were down-regulated. This phenomenon can be attributed to DNA hypermethylation, the amplification of H3K9me2 and/or the decrease in H3K9ac in the promoter region of the coding sites [60]. In soybean (*Glycine max*), increased levels of H3K4me3, H3K9ac and decreased levels of H3K9me2 coupled with DNA hypermethylation are the regulatory agents for the salinity responsive genes *Glyma11g0200*, *Glyma08g41450 and Glyma20g30840* [61]. Salinity stress is the main cause for the swelling up of roots in maize, and it has also been demonstrated that the cell-wall-related genes, namely, *ZmEXPB2* and *ZmXET1* are up-regulated with the simultaneous increase in the levels of H3K9ac [56].

The expression of cellulose expressing genes like *MYB54*, *CTL1*, *PGX3* are taken care of by the histone acetyltransferase of *Arabidopsis thaliana* (*AtGCN5*) and wheat (*TaGCN5*). The above-stated genes (*MYB54*, *CTL1*, *PGX3*) ease the way for H3K14 and H3K9 acetylation and thus maintain rigidity of the cell wall and tolerance to salinity [57]. In a diagonally contrasting study, it was demonstrated that HDA9 interacts with numerous stress-responsive genes, both abiotic/biotic and quells them directly [93,94]. These unfortunate genes include significant names such as Ethylene Response Factor (*EFR*, *ERF4/5/6/11*), kinase2 (*KIN2*), salt tolerance zinc finger (*STZ*). The composition of HDA9 is simple with a core histone deacetylase complex, consisting of HOS15 and PWR. HDA9 represses the above stress-related genes by adjusting histone methylation and histone acetylation [95]. An interesting case is noticed during salt tolerance in rice, where the histone deacetylase HDA1 performs a strange function. It represses the gene expression of the LATE EMBRYOGENESIS ABUNDANT PROTEIN1 (*LEA1*) and SALT OVERLY EXPRESSED (*SOS1*) by hindering the H3 acetylation path and linking up with INTERMEDIATE SPIKLET1 (*IDS1*) and TOPLESS-RELATED1 (*TPR1*) at the promoter sites of SOS1 and LEA1 [58]. Severe salt stress enriches the AGO2 proteins located on the BIG GRAINS3 (BG3) locus in turn stimulating the gene expression of BG3 by modulating the levels of H3K4me3 and H3K27me3 [96]. In rice, it has been experimentally proven that a protein complex made up of BCL-2-ASSOCIATED ATHENOGENE4 (*OsBAG4*), *OsSUVH7* and OSMYB106 controls and modulates the gene expression of *OsHKT1;5* as a response to extreme saline conditions [97]. In *Arabidopsis thaliana*, it has been reported that the SKB1 (floral initiator Shk1 kinase binding protein1) unites with H4R3 (Histone4 arginine3) symmetric demethylation (H4R3sme2) and have a combined reaction to salt stress. During severe stress, there is a drop in the level of H4R3sme2 and this happens due to the separation of SKB1 from the chromatin for the purpose to induce the stress-responsive genes. This entire process, in turn, increases the methylation of small nuclear ribonucleoprotein Sm-like4 (LSM4) [98]. In a detailed work regarding the expression and function of the *JMJ15* gene (*Arabidopsis thaliana* H3K4 demethylase gene), it was studied that the over-expression of this gene led to the stunted growth of plants with high lignin content in the stem tissues and amplified salt tolerance, while the knock-down mutants were severely salt sensitive. Transcriptomics of these mutant varieties revealed that the over-expressed variety supposedly down-regulated a plethora of genes with H3K4me3 and H3K4me2 markers. Overall, these experiments suggested that the amplified levels of JMJ15 protein might play a crucial role in governing the gene expression pattern of the salt-stress responsive genes which make the plant extremely tolerant to the above stress [99]. The histone H2B monoubiquitination (H2Bub) modulates the expression of the PROTEIN TYROSINE PHOSPHATASE1 (PTP1) and *MAP KINASE PHOSPHATASE* (*MKP*) group of genes that are compulsory for the depolymerization of stress-induced microtubule (MT) depolymerization and has an impact *on* the triggering of mitogen-activated protein kinase3 and 6 (MPK3, MPK6) [64]. AtMYB74, a transcription factor triggered to action during salinity stress, is fine-tuned by the decreased concentrations of 24-nt siRNAs and RdDM located at the promoter site [100].

To combat all types of stress, plants possess an adaptive trait called environmental stress memory. Here, the plant, upon exposure to primary stress, acquires memory and responds to the recurring stress events lightning fast. Proline accumulation is a marking phenomenon of higher plants for adaptation during various types of stresses. A proline biosynthetic enzyme Δ^1^-pyrroline-5-carboxylate synthetase 1(*P5CS1*) is expressed resulting in proline accretion. During recurrent phases of saline stress, this gene was more intensely induced and was dependent on subjection to light. So, proline accumulation which is salinity induced, is dependent on a memory gene and light signalling via HY5 is mandatory for such a response [62]. Castor (*Ricinus communis*) beans can grow in extreme saline soil in all proportions. This capacity is attributed to the MYB-associated transcription factor RADIALIS-LIKE SANT (RSM1), whose transcription is controlled by modifications in H3K4me3 and H3K27me3 [63]. Cytosine methylation is another important chromatin modification that is affected during salinity stress, which also regulates gene expression in numerous food crops such as rice, olive, wheat, barley etc. This gene expression is highly genotype and tissue-specific [85,101,102,103]. In wheat, high salinity induces cytosine methylation and this chromatin modification down-regulates the expression of some tissue-specific (in the root and shoot tissues) genes such as *Triticum aestivum HIGH-AFFINITY POTASSIUM TRANSPORTER2;1* (*TaHKT2;1*) and *TaHKT2;3* while on the other hand *TaHKT2;3* remains fully unaffected [101]. The magnitude of expression of the salt-stress-responsive genes in *Medicago tranculata* such as *WRKY*, *LEA*, *bZIP*, *KAT3*, *AP2/ERF* and *NAC* are related to the modified levels of cytosine methylation at the promoter sites [104].

## 4. Crosstalk between Chromatin Modification, Histone Modification, DNA(de-)Methylation and Non-Coding RNAs during Abiotic Stress-Induced Transcriptional Reprogramming

**Figure 3:**
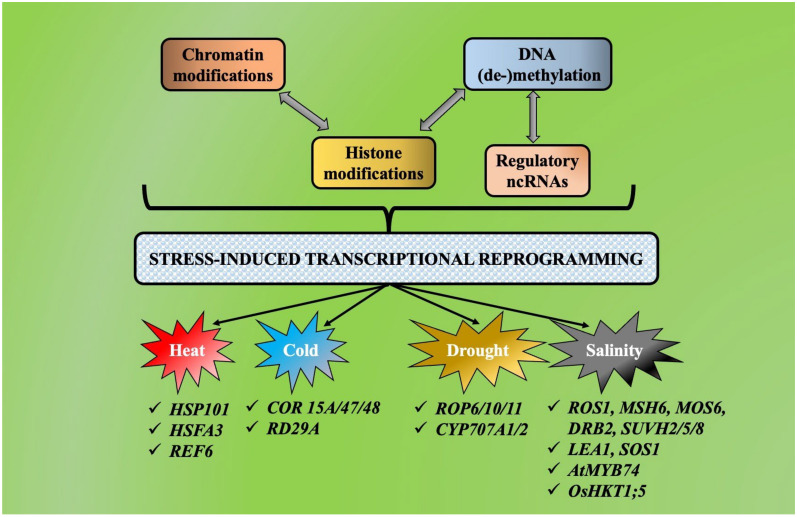
Crosstalk between chromatin modification, histone modification, DNA(de-)methylation and non-coding RNAs during abiotic stress-induced transcriptional reprogramming. Major epigenetic regulators such as chromatin remodelers, histone variants, DNA modifiers and ncRNAs interact with each other in a cooperative manner under abiotic stresses (heat, cold, drought, salinity) to portray a multilayered stress-induced epigenetic regulations in plants.

Plants carry out a harmonious interplay between various chromatin remodelers to manage multiple abiotic stresses, and a vivid picture has come up which throws light on these crosstalks linked with the abiotic-stress-induced transcriptional reprogramming. The discreet events of histone modification such as methylation, acetylation, ubiquitination and phosphorylation in plants are all interconnected and form a distinct network or web in stress management and control [4,105]. H3K4me3 and H327me3 are two bivalent markers with antagonising functions, and both mark the corresponding stress-responsive gene. This significant activity throws light on the pertinence of possible crosstalk between various modified histone proteins during transcriptional reprogramming in stress conditions [43,63]. The conjoint interplay between the diverse categories of epigenetic regulators, such as DNA methylation, histone modification, chromatin remodeling and ncRNAs, need to be dealt with in detail by the researchers and scientists to obtain a clear picture of what occurs during stress-induced transcriptional reprogramming [5,106]. Precisely three lines of crosstalk have been identified during transcriptional reprogramming induced by various abiotic stresses. They are (a) histone and chromatin modification, (b) DNA methylation and non-coding RNAs and (c) DNA methylation and histone modification (Figure 3).

It has been established that during transcriptional reprogramming, numerous chromatin-modifying proteins interconnect with histone modifiers or with functional chromatin marks [107]. In the universal model plant *Arabidopsis thaliana*, the chromatin remodeler BRM complex associates with the HD2C by repressing *HSFA3* and *HSP101* genes by eliminating H4K16ac [32]. While, on the other side, AGO1 interacts with the stress-related genes and binds to them with the help of small RNAs, SW1/SNF complexes to boost their cold-stress related responses [40]. A similar case is noticed in the case of rice where AGO2 links up with elevated and lowered levels of H3K4me3 and H3K27me4, respectively of the *BG3* gene to facilitate chromatin modification and aid in its expression to combat saline-stress [96]. A chromatin remodeling complex is built up by the PWR proteins by utilizing HOS15, HD2C/HDA9 and ABI4 to suppress the cold-responsive (*RD29A*, *COR15A/47/78*) or the drought-responsive genes (*CYP707A1/2*), respectively [76,84]. There is also a crosstalk between histone modifiers and transcription regulators, which fine-tune the chromatin dynamics and gene expression leading to altered nucleosome pattern at the transcriptionally active locations [12,107,108,109]. When *Arabidopsis thaliana* is under drought stress, H3/H4 deacetylation takes place to suppress the ROP gene and to succeed in this act, coordination between the TF MYB96 and the deacetylase HDA15 is absolutely necessary [53]. However in the case of heat stress, the heat-induced TF (HSFA2) turns on REF6, which is a functional H3K27me3 demethylase that controls the expression of the ROP gene, ultimately leading to heritable thermomemory in the forthcoming generations [33]. In rice, H3 deacetylation occurs at the gene loci of the salt-responsive genes (*LEA1*, *SOS1*), and this is brought about by the interaction of the transcriptional repressors (IDS1/TPR1) with HDA1 [109].

Activities such as chromatin organization, genome stability, (post-)-transcriptional regulation are regulated by small and long non-coding RNAs, which are characterized by their multifaceted roles in the above-mentioned activities [110,111]. Small non-coding RNAs (sncRNAs) aid in gene/locus-specific DNA methylation using the RdDM route and experimentations have established an association between histone modification and DNA methylation, the latter performing significant roles in processes such as chromosome interactions, mRNA processing regulations, silencing of transposons, transcriptional repression/activation [112,113]. In three rice cultivars with variable drought tolerance capacity, the sncRNAs are supportively associated with the hypermethylated regions which serve as solid evidence of an existing interplay between small RNA plentitude, gene expression and DNA methylation during the stress response [85]. In *Arabidopsis thaliana*, 24-nt siRNA accumulation is turned down in response to salinity stress, which is in turn linked with the exceedingly methylated *AtMYB74* gene, whose transcription is triggered via the RdDM route [100]. In soybean, uninterrupted saline stress causes genome-wide DNA methylation which corroborates with the fact of cooperative epigenetic regulation by the stress-responsive protein-coding genes and lncRNAs [114].

Plants have a tough and sturdy regulatory network that carries out transcriptional reprogramming to combat stress, and this is brought about by the phenomenon of crosstalk between histone modifications and DNA methylation [5,113]. A burning example of DNA methylations as well as numerous types of histone modifications (such as H3K9ac, H3K9me2, H3K4me3) are conjointly synchronized to carry out transcriptional activation/repression of *Glyma08g41450*, *Glyma11g02400*, *Glyma20g30840*, *SUVH2/5/8*, *ROS1*, *MSH6*, *APUM3*, *MOS6* and *DRB2* (salt-responsive genes) in soybean and *Arabidopsis thaliana* [60,61]. In rice, it has been observed that during salinity stress, the *OsHKT1;5* gene expression is majorly activated by the concerted action of a transcription complex SUVH7-BAG4-MYB106, mainly constructed of a DNA methylation reader combined with a chaperon regulator and a transcriptional regulator [97]. Even after so much investigation to establish scientifically the existence of all possible crosstalks between all epigenetic regulators, this subject still lacks a lot of clarity and deserves much more research and investigations.

## 5. Chromatin-Based Transcriptional Reprograming for Stress Priming

It has been observed that when plants are treated with mild stresses, it started showing enhanced response to subsequent stresses as compared to a non-treated plant [115]. This interesting phenomenon is known as ‘plant stress priming’, also known as ‘hardening’, which is initiated when environmental stresses act as a cue, with a chance of facing such severe stress in near future [116]. As there are multiple factors such as DNA methylation, chromatin remodeling and histone modifications that contribute to plant’s stress responses, it is evident that a process such as chromatin dynamics is actively involved in plant priming [117]. After stress adaptation, stress recovery is another crucial aspact that provides insights into how and when a plant’s stress memory is created/regulated and this recovery is facilitated by processes such as RNA metabolism, post-transcriptional gene silencing (PTGS), and RdDM [118]. Stress priming in response to treatments such as abscisic acid (ABA), methyl jasmonate, salisylic acid (SA, beta-aminobutyric acid (BABA) and stresses such as drought, cold and heat across multiple plant species has been listed in Table 3. Stress priming/stress memory can be transmitted between generations and the process is referred to as adaptive transgenerational plasticity [119]. This concept was first hypothesized by Jean-Baptiste Lamarck in his book Philosophie Zoologique in 19th century, where he discussed phenotypic traits acquired due to the surrounding environment in one generation could be transferred to future generations, thus making simple organisms into more complex ones overtime [120]. When the stress memory is only detectable in the first stress-free generation, it is termed as intergenerational memory, whereas if the memory effect is detectable in at least two consecutive generations of offspring, it is called transgenerational memory [121]. The maternal hyperosmotic stress memory in *Arabidopsis thaliana* relies on DNA methylation, and the epigenetic changes associated with it are conditionally heritable. It passes to the next generation through the female lineage because of excessive DNA glycosylation activity in male germline [122]. Similarly, the hyper accumulation of H3K4 methylation along with transcription factor HSFA2 in the memory-related loci is directly involved with heat stress memory in *Arabidopsis thaliana* [34]. Under transgenerational drought stress, *Arabidopsis thaliana* DNA methylome is stable [92] and is directly involved with transgenerational resistance by responding globally to diseases in prior generations [123]. Another study in *Arabidopsis thaliana* showed hypomethylation at the pericentromeric region can provide inheritable transgenerational quantitative disease resistance [124].

**Table 3:** List of stress priming in diferent species of plants.

Treatment/Stress	Target Species	Result	Reference
Salt	*Solanum lycopersicum*	Enhanced resistance against salt stress	[125]
SA/BABA	*Oryza sativa*	Improved tolerance against cold stress	[126]
SA	*Sinapis alba*	Improved tolerance against heat stress	[127]
SA/BABA	*Cucumis sativus*	Improved tolerance against cold stress	[126]
Cold	*Arabidopsis thaliana*	Vernalization response	[128]
SA	*Arabidopsis thaliana*	Improved tolerance against heat stress	[129]
BABA	*Arabidopsis thaliana*	Improved abiotic stress resistance	[130]
Osmotic/oxidative stress	*Arabidopsis thaliana*	Change in Ca^2+^ signals under osmotic stress	[131]
Dehydration	*Arabidopsis thaliana*	Improvement in retaining water	[132,133]
ABA	*Arabidopsis thaliana*	Greater sensitivity in stomatal opening triggered by lighting	[134]
Methyl jasmonate	*Nicotiana sylvestris*	Quick nicotine accumulation	[135]
SA	*Triticum aestivum*	Increased tolerance against salt	[136]
Drought	*Triticum aestivum*	Increased grain fill under drought	[137]
Salt	*Triticum aestivum*	Improvement in resistance against salt stress	[138]
Dehydration	*Zea mays*	Water-retention improvement	[139]
SA/BABA	*Zea mays*	Improved tolerance against cold stress	[126]

## 6. Concluding Remarks and Future Prospects

Plants are exposed to numerous stresses, both abiotic and biotic, from which they cannot escape at any cost. This review deals with the majority of the available research information on the changes that take place during chromatin dynamics and chromatin modifications, where histone modification is the ace player that assists the plants to resist, combat and survive environmental abuses such as severe heat, cold, drought salinity etc. Both [140] and [141] have concluded in their respective reviews that with the vast advancement of technology, there will be a deluge of datasets in the years to come helping researchers to create a detailed 3D picture of the plant transcriptomes and epigenomes and eventually fish out multiple master regulators responsible for chromatin folding and positioning, especially applicable for food and cash crops [142]. High-throughput methods such as high-throughput chromosome conformation capture (Hi-C) and chromatin interaction analysis by paired-end tag sequencing (ChIA-PET) have enabled researchers to explore complex chromatin interaction and organization [143]. In fact, fluorescent in situ hybridization (FISH) was the first microscopy-based technology to be used to study chromosomal organization in the nucleus and how it regulate gene expressions [144]. RNAs are known to influence local chromatin structure by interacting with DNAs at transcription sites (cis-acting) or distal sites (trans-acting) [145]. Several high-throughput methods such as chromatin-associated RNA sequencing (ChAR-seq) [146], GRID-seq [147] and mapping RNA genome interactions (MARGI) [148] have been developed to explore this type of RNA-DNA interaction across genomes. Chromosomes have been subdivided into self- interacting topologically associating domains (TADs) using Hi-C, but it is not clearly understood how they form. Technological advances have led researchers to use machine learning methods to elucidate chromatin-folding characteristics associated with TADs by coming up with novel logistic regression models [149].

Researchers from all over the world are working on ways and methods of facilitating plant stress resistance/tolerance by dissecting epigenetic regulation of the transcriptional stress memory response. Off late, transcriptional priming is a well-tried out application that takes place via chromatin modification of a few precise genes [52,150]. To carry out this process, a CRISPR-dCas9 system linked with either any transcriptional activator or histone acetyltransferase/methyltransferase can be used. ChIP assays have also proven efficient for the identification of histone modifications that actually carry out epigenetic regulation, but the process is not foolproof yet [4]. Chromatin-dynamics- based transcriptional regulation is an extremely necessary aspect in a plant’s life cycle for instant stress response and further memory response, which is still an enigma among plant scientists. Stress memory genes of different genres such as epigenetic memory, transcriptional memory or delayed memory indicate a major role of epigenetic markers in stress-related transcriptional memory [79]. Transcriptional reprogramming and transcriptional stress memory, are the two main processes that are carried out via epigenetic regulation. They also serve as the basis of the plant’s response, memory and these two steps are extremely crucial for crop improvement. Despite having hundreds of transcriptomic and histone modification data, there are still several major questions (for instance, after the stress signal perception, what are the kinetics of the changes in histone modification ? Do different cell types contribute towards different types of transcriptional reprograming due to their chromatin status? Is there a way to design epigenetic switches to control agronomical traits under stress situations? Is there a way to exploit epigenome modification to improve agricultural produce to feed the millions of the world?) that remained unanswered today. More and more research, analysis and minute detail are required in this field to have a clear idea of the interaction network between different epigenetic regulators, their modifications and how they synchronize transcriptional reprogramming and transcriptional stress memory to facilitate a successful plant response to abiotic stresses.

## Data Availability

Not applicable.
